# An Antibody-directed and Immune Response Modifier-augmented Photothermal Therapy Strategy Relieves Aging via Rapid Immune Clearance of Senescent Cells

**DOI:** 10.14336/AD.2023.0628-1

**Published:** 2024-04-01

**Authors:** Jiamei Han, Judun Zheng, Qian Li, Huanle Hong, Jing Yao, Jiao Wang, Robert Chunhua Zhao

**Affiliations:** ^1^School of Environmental and Chemical Engineering, Shanghai University, Shanghai 200444, China.; ^2^School of Life Sciences, Shanghai University, Shanghai 200444, China.; ^3^Institute of Basic Medical Sciences Chinese Academy of Medical Sciences, School of Basic Medicine Peking Union Medical College, Center of Excellence in Tissue Engineering, Chinese Academy of Medical Sciences, Beijing Key Laboratory of New Drug Development and Clinical Trial of Stem Cell Therapy (BZ0381), Beijing, China.; ^4^Cell Energy Life Sciences Group Co. LTD, Qingdao, Shandong, China.; ^5^Molecular Diagnosis and Treatment Center for Infectious Diseases, Dermatology Hospital, Southern Medical University, Guangzhou, Guangdong, China.

**Keywords:** Cellular senescence, photothermal therapy, immune response, β2-microglobulin, anti-aging biomaterials

## Abstract

Cellular senescence is an irreversible and multifaceted process inducing tissue dysfunction and organismal aging, and thus the clearance of senescent cells can prevent or delay the onset of aging-related pathologies. Herein, we developed an augmented photothermal therapy strategy integrated with an antibody against β2-microglobulin (aB2MG) and an immune adjuvant imiquimod (R837) to effectively accelerate senescent cell apoptosis and clearance under a near-infrared light. With this strategy, the designed CroR@aB2MG enables the targeting of senescent cells and the application of photothermal therapy concomitantly, the initiation of immune clearance subsequently, and finally the realization of protective effects against senescence. Our results showed that the photo-induced heating effect caused senescent cells to quickly undergo apoptosis and the synchronous immune response accelerated the clearance of senescent cells *in vitro* and *in vivo*. Therefore, this photoactivated speedy clearing strategy may provide an efficient way for the treatment of senescence-related diseases by eliminating senescent cells with biomaterials.

## INTRODUCTION

Senescent cells exhibit a state of irreversible cell cycle arrest and dysfunction [1], which are closely linked to numerous senescence-related diseases, such as Alzheimer’s disease [2], diabetes [3], and cancer [4]. And the development of senescence-associated secretory phenotype (SASP) in senescent cells accelerates the onset of aging and aging-related diseases [5]. Thus, the clearance of senescent cells from the tissue has been suggested as a promising strategy to prevent aging-related diseases including cancer [6]. Senescent cells can be seen as a highly important immunogenic target [7], owning to their susceptibility to the immune surveillance by the immune system to regulate senescent cell number [8]. Several researchers have revealed that the immune clearance enabled the clearance of senescent cells from the tissue with excellent biosecurity, delaying the process of aging or cancer [9-11]. However, treatments of this kind can be affected by immunodeficiency or limited clearance capacity of the immune system. Therefore, it is not only challenging to remove the overburdening aging cells, but also demanding to prevent chronic inflammation caused by some highly viable senescent cells [12]. We propose that developing an efficient strategy for clearing senescent cells is essential to delay aging.

**Figure 1.**
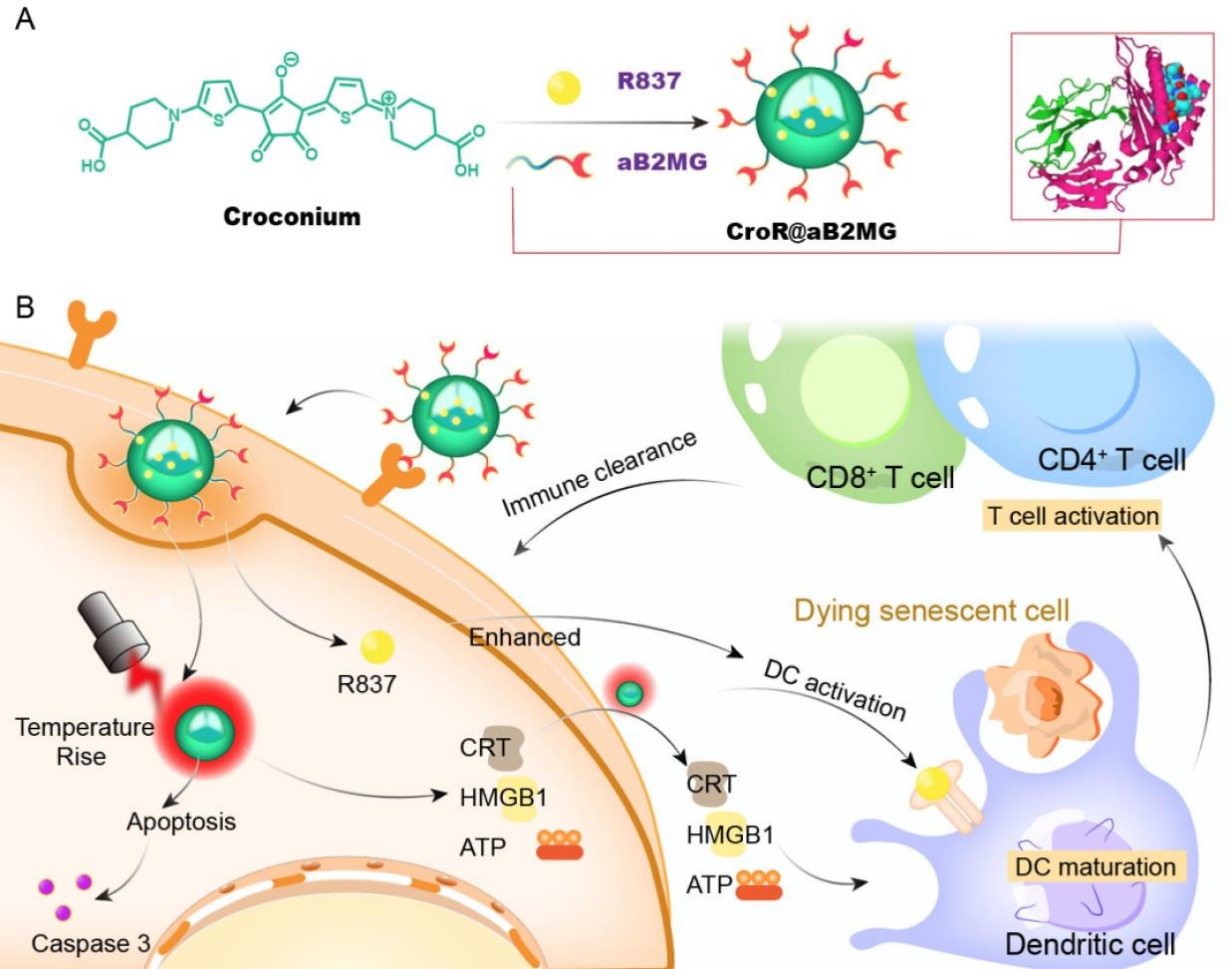
**The schematic diagram of clearance of photo-responsive senescent cells by CroR@aB2MG-integrated PTT**. **(A)** The synthesis of CroR@aB2MG. The Cro dye is enclosed by aB2MG, forming Cro@aB2MG. R837 is physically adsorbed to the hydrophobic pocket of Cro@aB2MG, generating the full package of CroR@aB2MG nanoparticles. **(B)** The proposed mechanism of senescence cell clearance by the combination of PTT-mediated cell apoptosis and CroR@aB2MG-induced immune activation. The CroR@aB2MG nanoparticles anchor on senescent cells with the direction of aB2MG. The Cro dye exerts photothermal effects under NIR irradiation and induces immunogenic apoptosis. These cells generate and release DAMPs, such as calreticulin (CRT), high-mobility group box1 (HMGB1) and ATP, which promote the maturation of DCs. Meanwhile, the immune response modifier R837 further activates DCs and T cells, which assists in the presentation of dying senescent cells to CD8^+^ killer T cells and the realization of immune clearance. A sub-population of CD4^+^ T cells act as the effector memory T cells to facilitate further immune clearance.

Photothermal therapy (PTT), a non-invasive technique with excellent spatiotemporal selectivity, has been widely used to induce the apoptosis of damaging cells, such as cancer cells [13], which are sensitive to temperature under light irradiation[14]. The heat-induced cell death and damage promote the production of damage associated molecular patterns (DAMPs) [15], which can activate either the innate immune system or the adaptive immune system to enable immune clearance [16]. One of the biggest challenges of immune responses induced by PTT is the development of highly efficient and biocompatible light-absorbers [17]. To minimize the side effects, the absorption spectrum of photothermal agents with good penetrability should fall in the near-infrared (NIR) range (700-1100 nm), in which case biological molecules including hemoglobin and melanin are optically transparent [18]. Several inorganic nanomaterials such as carbon and gold nanoparticles, possess certain photophysical properties, including strong photothermal conversion efficiency and high photothermal stability, and have been widely used as antitumor and antibacterial materials[19]. However, inorganic nanoprobes are hard to degrade and thus are potentially toxic. Recently, organic NIR molecules with photothermal effects, such as heptylamine and phthalocyanines, have been developed to accelerate the apoptosis of detrimental cells, for example, cancer cells [20, 21]. These photothermal dyes including the FDA-approved indocyanine green (ICG) dye are either subjected to degradation due to the reactive oxygen species (ROS) generated under NIR irradiation, or limited in application due to their intrinsic properties such as poor water solubility, self-aggregation and short circulation half-life[22]. Among other organic molecule-based photothermal agents, the croconium (Cro) dyes, also known as croconaine dyes, have been widely studied due to their intense NIR absorption and high photostability [23]. Particularly, the Cro dyes are easy to synthesize and are structurally modifiable to improve their water solubility, microenvironment responsiveness and photothermal effects [24, 25].

Herein, we designed and developed an augmented PTT strategy based on the recognition of senescent cells through an antibody against β2-microglobulin (aB2MG), the subsequent induction of apoptosis via NIR irradiation with the Cro dye, and the simultaneous immune activation by an immune response modifier imiquimod (R837)[26] (Fig. 1). Specifically, the abundant expression of β2-microglobulin (B2MG) on the surface of senescent cells allows for high affinity of the designed CroR@aB2MG nanoparticles, the 808 nm-light-induced heating effect quickly directs senescent cells to apoptosis and R837 stimulates the immune responses to clear senescent cells. We demonstrate that a large number of senescent cells experienced apoptosis with the treatment of CroR@aB2MG under an NIR light, which generated immunological effects represented by dendritic cell maturation, T cell recruitment, cytokine secretion and effector memory cell activation, implying great potential to guide the immune system to attack more senescent cells. As the combination of CroR@aB2MG and NIR irradiation enables efficient targeting, killing and elimination of senescent cells, the photoactivated speedy clearing strategy is a promising candidate for the treatment of senescence and senescence-related diseases via eliminating senescent cells with highly efficient biomaterials.

## MATERIALS AND METHODS

### Instruments used for material characterization

Proton nuclear magnetic resonance (^1^H NMR) measurements were performed with an Avance II 400 MHz NMR spectrometer (Bruker, USA). Molecular weights were determined by electrospray ionization mass spectrometry (ESI-MS) (Agilent, USA). The physical features of nanoparticles were observed with a JEM-1400 Flash electron microscope (JEOL, Japan) at 120 kV. Absorption spectra were obtained with a NanoDrop 8000 spectrometer (Thermo Fisher Scientific, USA). Fluorescence imaging was performed using a laser scanning confocal microscope (Zeiss, Germany).

### Material

Methyl isonipecotate (≥70%, Sigma, USA), thiophene-2-thiol (≥99%, Sigma, USA), ethyl acetate (≥99.8%, Energy Chemical, China), croconic acid (>95%, Aladdin, China), n-butanol (≥99%, Aladdin, China), toluene (98%, Yishi Chemical, China), N-hydroxysuccinimide (NHS, 98%, Aladdin, China), 1-ethyl-3-(3-dimethylaminopropyl) carbodiimide hydrochloride (EDC, Aladdin, China), imiquimod (R837, 99%, Meryer, China), aB2MG (ABclonal, China), 4,6-biphenyl-2-phenylindole (DAPI, Beyotime, China). For immunostaining experiments, the following antibodies were used: rabbit anti-forkhead box O4 (FOXO4) (A17978, ABclonal, China), rabbit anti-p16^ink4a^ (A0262, ABclonal, China), rabbit anti-p21CIP1 Rabbit pAb (A1483, ABclonal, China), rabbit anti-lamin B1 (A16909, ABclonal, China), rabbit anti-p53 (A0263, ABclonal, China), rabbit anti-decoy receptor 2 (DcR2) (A6136, ABclonal, China), mouse anti-glyceraldehyde 3-phosphate dehydrogenase (GAPDH) (AC002, ABclonal, China), goat anti-rabbit IgG H&L Alexa Fluor^®^ 594 (ab150080, Abcam, UK), HRP goat anti-rabbit IgG (H+L) (AS029, ABclonal, China) and HRP goat anti-mouse IgG (H+L) (ab205719, Abcam, UK).

### Synthesis of the Cro dye

4.30 g (30.0 mmol) of methyl isonipecotate and 2.32 g (20.0 mmol) of thiophene-2-thiol were mixed in 20 mL of toluene and were refluxed for 2 h. After cooling to room temperature, the reaction product was filtered through a silica gel and washed with ethyl acetate, which resulted in a pale-yellow solid of methyl 1-(thiophen-2-yl) piperidine-4-carboxylate (compound a) with a yield of 59%.

Next, 0.45 g (2.0 mmol) of compound a was dissolved in 10 mL of 0.5 M sodium hydroxide and was refluxed for 1 h. The reaction product was acetoxylated with 10% acetic acid to generate [1-(thiophen-2-yl) piperidine-4-carboxylic acid] (compound b) as a white precipitate with a yield of 68%.

Subsequently, 0.142 g (1.00 mmol) of croconic acid and 0.422 g (2.00 mmol) of compound b were mixed in n-butanol and toluene (30 mL, 1:1) and were refluxed for 1 h. After cooling to room temperature, the reaction product was filtered, washed with methanol, and dried in vacuum, generating the Cro dye as a black solid with a yield of 51%. The molecular structure of the Cro dye was determined as ^1^H-NMR (600MHz, d6-DMSO) δ12.40 ppm (s, 2H), 8.35 ppm (s, 2H), 7.05-7.23 ppm (m, 2H), 4.01 ppm (d, J= 9.0 Hz, 4H), 3.68 ppm (t, J= 6.0 Hz, 4H), 2.67 ppm (s, 2H), 2.05-1.95 ppm (m, 4H), 1.74-1.65 (m, 4H).

### Preparation of CroR@aB2MG nanoparticles

CroR@aB2MG nanoparticles were obtained by self-assembly of R837 with the synthesized Cro dye and aB2MG. Briefly, the Cro dye (15.0 mg, 0.28 mmol), NHS (48.3mg, 0.42 mmol), and EDC (65.1 mg, 0.42 mmol) were dissolved in dimethyl sulphoxide (DMSO), and the mixture were stirred for 4 h. The aB2MG (25 mg) was added into the above solution, which was then stirred overnight. Free Cro was removed by dialysis with deionized water and the Cro@aB2MG nanoparticles were obtained through free-drying. For preparing CroR@aB2MG, the Cro@aB2MG nanoparticles (25 mg) were redissolved in phosphate-buffered saline (PBS, 2 mL). R837 (5 mg) was dissolved in 30 μL DMSO and added dropwise into the Cro@aB2MG solution. The mixture was stirred overnight in dark and subjected to centrifugation and ultrafiltration to obtain the CroR@aB2MG nanoparticles. The final product was stored at 4 °C.

### Ultraviolet visible (UV-vis) spectroscopy

Test solutions were prepared by adding 2 mL of the sensor stock solution into a cuvette. The absorption spectra were recorded at 300 to 900 nm.

### Determination of photothermal properties

The thermal effect of the CroR@aB2MG solution under 808 nm laser irradiation of different power densities (0.5, 0.75, 1.0, 1.5 W/cm^2^) was determined. The temperature rise of pure water and the CroR@aB2MG solution in the photothermal heating process was measured by an infrared thermometer.

### Calculation of the photothermal conversion efficiency

The CroR@aB2MG solution (1.0 mg/mL) was irradiated with an 808 nm laser (1.0 W/cm^2^) until the solution temperature stabilized. Next, the solution was naturally cooled to ambient temperature after the laser radiation was removed. The light-to-heat conversion efficiency (η) of CroR@aB2MG nanoparticles was calculated using the following equation:

$$\eta =\frac{\mathrm{hs}(T_{\max}-T_{\mathrm{surr}})-Q_{0}}{I(1-10^{-A})}$$

(T_max_-T_surr_) is the temperature change of the CroR@aB2MG solution before and after the temperature in the surrounding environment (T_surr_) reaches the maximum temperature (T_max_). Q_0_ refers to the heat absorbed by the solvent and the container. I is the power density of the laser. A is the absorbance of CroR@aB2MG nanoparticles at 808 nm.

### Animals

In this study, C57BL/6 female mice of 6-8 weeks were used. All mice were purchased from Shanghai Experimental Animal Co., Ltd. (Shanghai, China) and were kept free of pathogens in accordance with the international animal care guidelines. All animal experiments were performed under constant humidity (65%) and constant temperature (22±2°C), with the approval of the Animal Protection Committee of Shanghai University.

### Cell culture

Normal human dermal fibroblast (NHDF) cells, human embryonic kidney 293T cells (293T) and Hela cells (an immortal human cell line) were purchased from ATCC (American Type Culture Collection) and maintained in Dulbecco's modified Eagle’s Medium (Thermo Fisher Scientific, USA) supplemented with 10% fetal bovine serum (FBS), penicillin/streptomycin, and 0.05% glutamine. Cells were maintained at 37°C with 5% CO_2_ and 3% O_2_. To induce senescence, cells were incubated with 250 μm H_2_O_2_ for 2 h, or treated twice with 0.1 mM doxorubin (DOX) with a 2-day interval and were assayed 7 days later. Aging was determined by the staining of senescence-associated β-galactosidase (SA-β-GAL) and the evaluation of changes in cell morphology.

For culturing dendritic cells (DCs) *in vitro*, female adult mice were anesthetized with intraperitoneal injection of chloral hydrate and sacrificed to harvest bone marrow cells. In a sterile dissection hood, the femur and tibia were collected. Muscles and adipose tissues were removed. The epiphyses on both sides of the bones were cut off and the bone marrow cavity was rinsed with PBS. Bone marrow cells in PBS were collected and pipetted repeatedly to obtain a single cell suspension. Then the red blood cell lysis buffer was added, and the cell suspension was pipetted for 3-4 min, followed by the addition of 10 times the volume of PBS to terminate the lysis reaction. The cells were washed once with PBS, resuspended in RPMI-1640 medium (Thermo Fisher Scientific, USA) containing 10 ng/mL mouse GM-CSF (Thermo Fisher Scientific, USA), 5 ng/mL mouse IL-4 (Thermo Fisher Scientific, USA) and 10% FBS, and cultured for 7 days to obtain immature DCs.

### Cell viability assay

DOX-induced senescent NHDF cells, H_2_O_2_-induced senescent 293T cells, and DOX-induced senescent Hela cells were incubated with different concentrations of CroR@aB2MG (0.125, 0.25, 0.5 and 1.0 mg/mL) for 24 h. The number of live cells was determined with a CCK-8 kit (Beyotime, China). The optical density (OD) was measured at a wavelength of 450 nm. The apoptotic rates of senescent cells after the treatments of NIR irradiation in combination with different nanoparticles were measured through flow cytometry with an Annexin V-FITC/PI (propidium Iodide) kit (C1062-S, Beyotime, China) or a caspase 3 antibody (C1077S, Beyotime, China). The NIR irradiation was performed with an 808nm laser at 1.0 W/cm^2^ for 10 min and the photothermal agents tested were Cro, Cro@aB2MG or CroR@aB2MG. Cells incubated with PBS or CroR@aB2MG without NIR irradiation and cells receiving NIR irradiation without photothermal agents were also included for inter-group comparison.

### Western blotting

NHDF cells (1.0 × 10^6^) were collected, and total proteins were extracted with RIPA (Radioimmunoprecipitation) lysis buffer (Beyotime, China). The protein lysates were then separated by SDS-PAGE and transferred to nitrocellulose filter membranes. The membranes were incubated with primary antibodies rabbit anti-p53 (1:1000), rabbit anti-p16^ink4a^ (1:1000), rabbit anti-DcR2 (1:1000), rabbit anti-FOXO4 (1:1000), or mouse anti-GAPDH (1:10000) and then with HRP goat anti-rabbit IgG (H+L) or HRP goat anti-mouse IgG (H+L) secondary antibodies (1:1000). The immune-positive bands were visualized using an Odyssey scanner (LI-COR Biosciences, USA). The band densities of p53, DcR2, p16^ink4a^, and FOXO4 were quantified in ImageJ and normalized against those of GAPDH, representing their relative protein expression levels respectively.

### Analysis of DCs surface marker expression in vitro

DCs cultured *in vitro* were incubated with different nanoparticles (Cro, Cro@aB2MG or CroR@aB2MG) of 0.5 mg/mL under NIR irradiation. After the treatments, cells were collected and subjected to flow cytometry with PE anti-mouse CD80 (104707, BioLegend, China) and APC anti-mouse CD86 (105011, BioLegend, China). The stained cells were quantified with the Flow Jo software.

### The analysis of immune responses and the anti-aging effects of CroR@aB2MG in induced senescent mice

Mice of 26 weeks were intraperitoneally injected with two doses of DOX at a dosage of 10 mg/kg with a 5-day interval. Five days after the second dose, mice received an intravenous injection of 0.5 mg/mL Cro, Cro@aB2MG or CroR@aB2MG. Two days later, the mice were exposed to NIR irradiation. Another three groups of mice were injected with PBS without NIR irradiation, were injected with CroR@aB2MG without NIR irradiation or received NIR irradiation without the administration of any photothermal agent.

To evaluate the generation of mature DCs and the proportions of different types of T-cells *in vivo*, some mice were euthanized for the collection of inguinal lymph nodes to determine the percentages of CD80^+^CD86^+^ DCs. The spleens of some other mice were collected to determine the proportions of cells co-expressing TNF-α (tumor necrosis factor-α) and IFN-γ (interferon-γ), CD4^+^ T cells, CD8^+^ T cells and different types of memory T cells. Antibodies PE anti-mouse CD80 (104707, BioLegend, China), APC anti-mouse CD86 (105011, BioLegend, China), PE anti-mouse CD4 (100407, BioLegend, China), APC anti-mouse CD8a (100711, BioLegend, China), PE/Cyanine7 anti-mouse TNF-α (506323, BioLegend, China), PerCP/Cyanine 5.5 anti-mouse IFN-γ (505821, BioLegend, China), PE anti-mouse/human CD44 (103007, BioLegend, China), and PerCP/Cyanine 5.5 anti-mouse CD62L (104431, BioLegend, China) were used for flow cytometry. The BD Cytofix/Cytoperm^TM^ fixation/permeabilization kit (BD Biosciences, USA) was used for fixation and permeabilization. Results were analyzed with the Flow Jo software. The remaining mice were evaluated 35 days later to compare the renal senescence and fur appearance across different treatment groups. For determining the renal senescence, the mice were euthanized for the collection of kidneys. The kidneys were fixed with 4% paraformaldehyde (PFA, Sigma, USA), and cut to obtain 30 µm sections with a vibratome (Invitrogen, USA). The sections were subjected to fluorescent immunolabeling to determine the expression of p53 and lamin B1.

### Immunofluorescence assay

For immunocytochemistry experiments, cells were grown on glass coverslips and treated as indicated before being fixed with 4% PFA. Subsequently, the cells were washed with Tris buffer (TBS) and permeabilized in 0.25% Triton X-100 for 5 min. To reduce the background staining, the cells were quenched with 50 nM glycine in TBS for 10 min, and were blocked with 1% BSA or 0.2% gelatin/TBS for 30 min. Next, microdroplets (30 μL) containing primary antibody dilutions were placed on the sealed membrane in a dark humid chamber. The coverslips were placed in front of the drop and incubated overnight at 4°C. The next day, a small amount of gelatin/TBS was added to the underside of the coverslips. Then the coverslips were lifted up and returned to the 24-well plate. After being washed 3 times with 1 mL of 0.2% gelatin/TBS for 20 min each, the coverslips were incubated with the goat anti-rabbit IgG H&L Alexa Fluor^®^ 594 secondary antibody (1:300) at room temperature for 1 h. After the incubation, the coverslips were washed 3 times (10 min each) with 1 mL of 0.2% gelatin/TBS, once with regular TBS, and were then incubated with a solution containing DAPI before being mounted on slides. For immunohistochemistry experiments, the fixed tissue sections of mouse kidney were mounted onto glass slides and subjected to immunolabeling following steps described above. For all the immunofluoresence staining experiments, cells or kidney sections from the same batch or mouse were also stained with only secondary antibodies as the negative control, which were used to determine the specificity of primary antibodies and the background noise level for taking confocal images.

**Figure 2.**
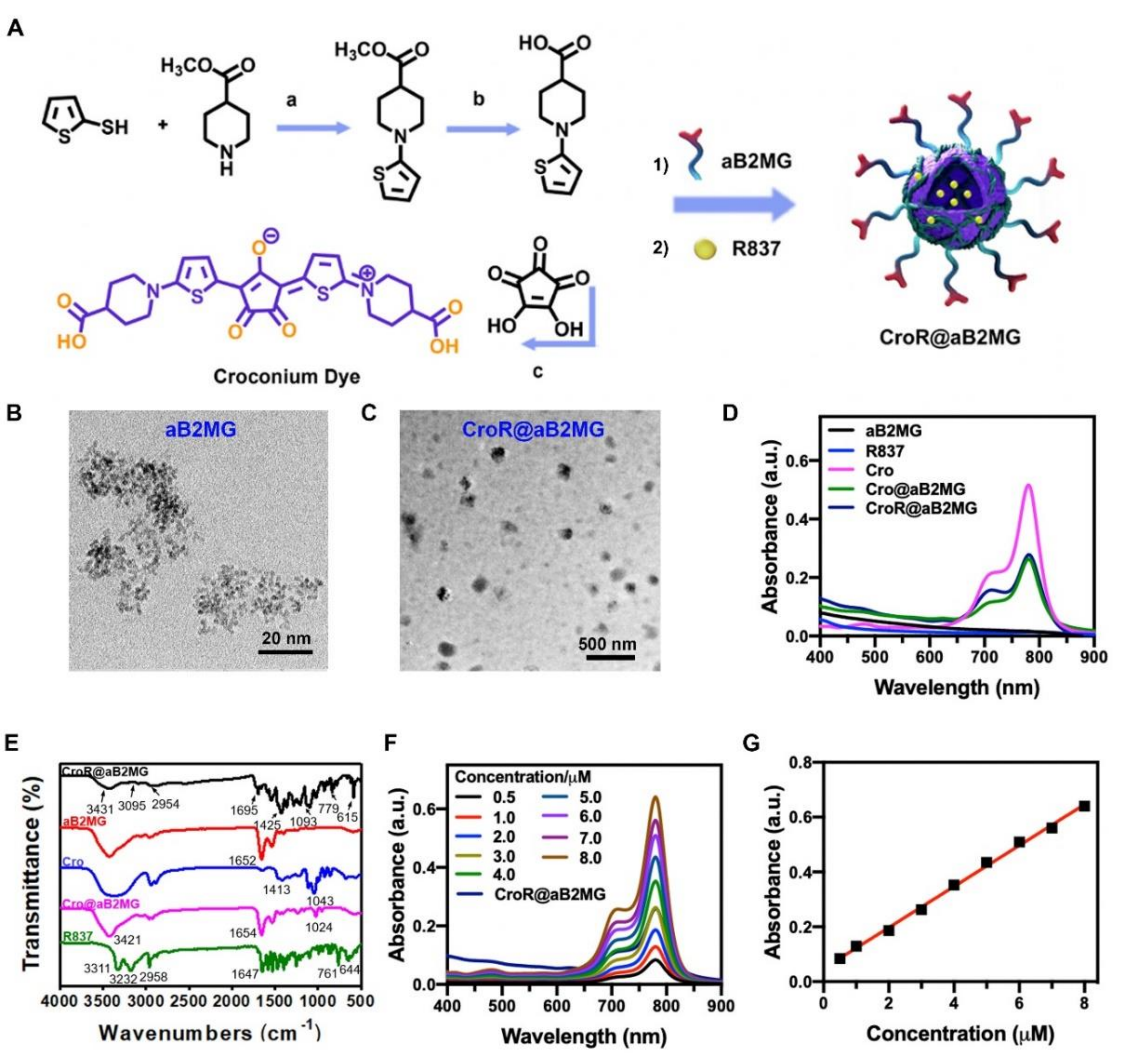
**The structure and characterization of CroR@aB2MG nanoparticles**. **(A)** The schematic diagram of the preparation process of CroR@aB2MG. (a) Methyl isonipecotate and thiophene-2-thiol were refluxed together. (b) The reaction product from (a) was deprotected in an alkaline solution. (c) The reaction product from (b) underwent condensation reaction with croconic acid, generating the Cro dye (thiophene-croconaine). An anti-B2MG antibody wrapped in Cro dye and R837, resulting in the formation of CroR@aB2MG nanoparticles. (B and C) TEM images showing the size and distribution of aB2MG and CroR@aB2MG nanoparticles. Scale bars, 20 nm and 500 nm. **(D)** The UV-vis spectra of aB2MG, R837, Cro, Cro@aB2MG and CroR@aB2MG. **(E)** The FT-IR spectra of aB2MG, R837, Cro, Cro@aB2MG and CroR@aB2MG. **(F)** The UV-vis spectra of CroR@aB2MG and the Cro dye with the concentration ranging from 0.5 to 8.0 µM. **(G)** The standard absorbance curve of Cro at 779 nm.

### Statistical analysis

All quantitative data are shown as mean±SEM in the figures. The normality of data was determined with the Shapiro-Wilk test before the test of difference significance. For data that followed the normal distribution, Student’s t-test was performed for two groups, and the one-way ANOVA (analysis of variance) with Tukey's post hoc test for more than two groups to determine the significance of difference between different groups. The significance levels were set at *p < 0.05, **p < 0.01, ***p < 0.001, and ****p < 0.0001. The data were analyzed, and the statistical graphs were drawn with GraphPad Prism 9.0.

## RESULTS

### Synthesis and characterization of CroR@aB2MG

The Cro dye was synthesized in three steps, enclosed by aB2MG and mixed with R837, generating CroR@aB2MG nanoparticles (Fig. 2A). Specifically, Cro and aB2MG were connected through the amidation reaction between the carboxyl groups in Cro and the amino groups in aB2MG. R837 was then adsorbed into the hydrophobic pocket of the Cro@aB2MG via the hydrophobic-hydrophobic interaction between Cro and R837. The molecular structure of the Cro dye was determined by ^1^H NMR (Supplementary Fig. 1), and its molecular weight was measured to be 530.623 by ESI-MS (Supplementary Fig. 2). The morphology and size (89 ± 5.34 nm) of CroR@aB2MG were verified by transmission electron microscopy (TEM) shown in Fig. 2C. The UV-vis spectra confirmed the successful construction of the nano-platforms as the inherent absorption peak of the Cro dye was still detectable in the Cro@aB2MG and CroR@aB2MG packages (Fig. 2D). In an agarose gel after electrophoresis, CroR@aB2MG moved further toward the positive electrode than aB2MG, because of the negative charges from the free carboxylates in the Cro dye (data not shown). The CroR@aB2MG showed a different zeta potential from that of Cro, aB2MG, R837, or Cro@aB2MG (Supplementary Fig. 3). The Fourier transform infrared (FT-IR) spectroscopy further distinguished the nanoparticles based on the thiophene structure in Cro and the benzene ring structure in R837. As shown in Figure 2E, the vibration peaks of C-S-C could be found at 1043, 1024 and 1093 cm^-1^, indicating the thiophene structures in Cro, Cro@aB2MG and CroR@aB2MG. The characteristic vibrational modes corresponding to the C-H and C-C stretches in the aromatic ring of benzene were clearly observed in the FT-IR spectra of CroR@aB2MG (at 3431 and 1425 cm^-1^ respectively) and R837 (at 3311 and 1510 cm^-1^ respectively) (Fig. 2E). The additional peaks between 400 and 1500 cm^-1^ referred to the C-H vibrations in the fingerprint region of an FT-IR spectrum for benzene, represented by the absorption at 779 and 615 cm^-1^ for CroR@aB2MG, and that at 761 and 644 cm^-1^ for R837. The peaks at 1695, 1652, 1654 and 1647 cm^-1^ were attributed to the C-N bonds common in Cro, R837 and proteins. And the mass ratio of the Cro dye to CroR@aB2MG was determined to be ~11.26% according to a standard absorbance curve of Cro (Fig. 2F and 2G).

### Optical properties and photothermal effects of CroR@aB2MG

**Figure 3.**
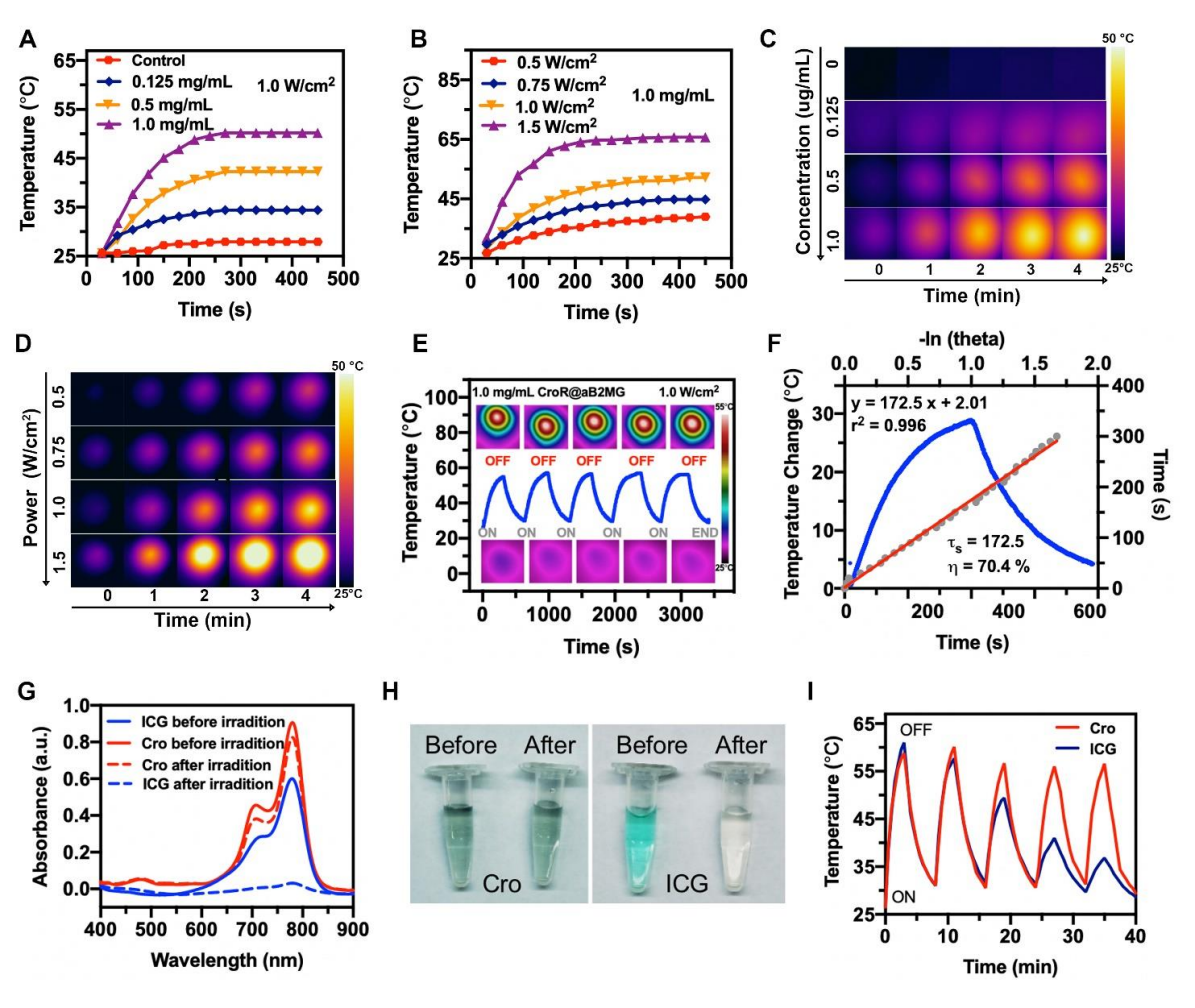
**The photothermal properties of CroR@aB2MG nanoparticles**. **(A)** Photothermal heating curves of CroR@aB2MG nanoparticles of different concentrations under 808-nm laser irradiation (1.0 W/cm^2^). The control was pure water. **(B)** Photothermal heating curves of CroR@aB2MG nanoparticles (1.0 mg/mL) under 808-nm laser irradiation of different power densities. (C and D) Thermal images of CroR@aB2MG nanoparticles corresponding to (A) and (B). **(E)** Photothermal heating/cooling curves and thermographic images of CroR@aB2MG nanoparticles (1.0 mg/mL) undergoing six cycles of 808-nm laser irradiation (1.0 W/cm^2^). **(F)** Plot of cooling time versus temperature change showing the photothermal effect of CroR@aB2MG. The -ln(theta) in the upper abscissa represents the negative natural logarithm of the driving force temperature. The time constant for heat transfer(τ_s_) and the photothermal conversion efficiency(η) were calculated to be 172.5s and 70.4% respectively. **(G)** The UV-vis spectra of Cro and ICG before and after the irradiation by an 808-nm laser at a power density of 0.5 W/cm^2^. **(H)** Representative photos of Cro and ICG solutions before (left) and after (right) the laser irradiation in (G). **(I)** Temperature curves of the Cro (1.0 mg/mL) and ICG (1.0 mg/mL) solutions undergoing 5 cycles of irradiation by an 808-nm laser at a power density of 1 W/cm^2^. Each irradiation cycle lasted 3 min.

The Cro dye, as a photothermal agent not only possesses high photothermal conversion efficiency, but also maintains excellent stability because it cannot be consumed through radiative relaxation pathways such as electron transfer or intersystem crossing [27]. In CroR@aB2MG solutions of different concentrations, an 808 nm diode laser was used to irradiate at an intensity of 1.0 W/cm^2^, and the photothermal heating curves are shown in Fig. 3A. As expected, the temperature of the CroR@aB2MG solution increased with time and began to stabilize after 5 min. The concentration of CroR@aB2MG is related to the heating rate and the plateau temperature. When the concentration of CroR@aB2MG was 0.5 mg/ mL, the temperature of the solution reached 43 °C after being irradiated for 5 min, which was enough to kill aged cells (Fig. 3A). Under the same experimental conditions, the temperature of pure water increased by 5 °C, indicating that the laser irradiation alone cannot produce sufficient photothermal effect, and the temperature increase of the CroR@aB2MG solution was mainly attributed to the photothermal conversion of the NIR absorption of CroR@aB2MG. Next, the temperature of the CroR@aB2MG solution was monitored at different powers. The temperature of 1 mg/mL CroR@aB2MG under a laser power of 0.75 W/cm^2^ rose to 45 °C after 5 min, which was also sufficient to kill senescent cells (Fig. 3B). Thus, a specific temperature of the CroR@aB2MG solution can be obtained by adjusting either the CroR@aB2MG concentration or the laser power (Fig. 3C, 3D). Thermal cycling experiments were performed and CroR@aB2MG exposed to repeated heating and cooling cycles (808 nm, 1 W/cm^2^) displayed excellent photothermal stability (Fig. 3E). The photothermal efficiency of CroR@aB2MG was measured within the range of temperature rising till the maximum and was calculated to be 70.4% (Fig. 3F). Next, we compared the photothermal stability and efficiency of Cro and ICG. A 20-min continuous NIR irradiation bleached ICG completely and had minimal effects on Cro (Fig. 3G, 3H). Consistently, compared with Cro, the maximum heating temperature generated from ICG showed a significant decline with the number of NIR irradiation cycle increasing (Fig. 3I). These observations confirm that the Cro dye is more stable and efficient than the traditional ICG dye in the process of photothermal conversion.

### Apoptosis of senescent cells targeted by CroR@aB2MG

**Figure 4.**
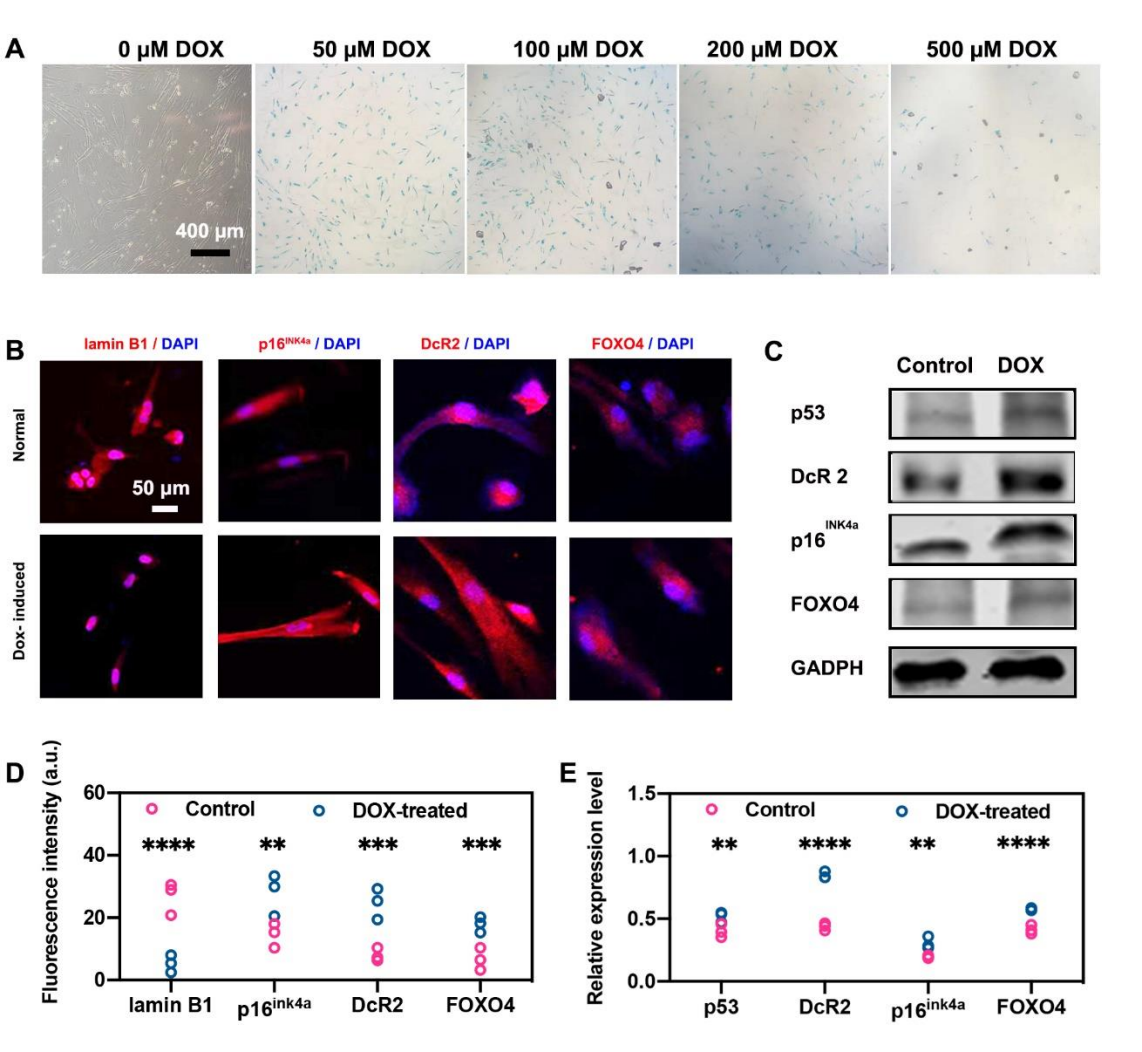
**The establishment of DOX-induced senescent cell model**. **(A)** SA-β-GAL staining was used to label senescent NHDF cells 7 days after treatments with DOX of different concentrations. (B and D) Representative confocal microscopy images and quantification of fluorescence intensities of immnolabeling for lamin B1, p16^ink4a^, DcR2 and FOXO4 in control and DOX-treated NHDF cells. The nucleus was stained with DAPI. For (D), n=3 batches of cells. For each protein in each batch of cells, 3 regions of interest with roughly the same numbers of cells on a coverslip were photographed and quantified for fluorescence intensities as 3 technical replicates. The intensity values were averaged to obtain a single data point as the expression level of the target protein in one biological replicate. The 3 batches of cells are regarded as 3 biological replicates. (C and E) Representative western blots and densitometric analysis of p53, DcR2, p16^ink4a^ and FOXO4 in NHDF cells without or with the induction of DOX. GAPDH was the loading control. The relative expression level was calculated by normalizing the band densities of p53, DcR2, p16^ink4a^ and FOXO4 against those of GAPDH. For (E), n=3 batches of cells, indicating 3 biological replicates. For (D) and (E), the significance of differences in the expression level between the control group and DOX-treated group was determined through Student’s t-test, and shown as **p <0.05, ***p <0.001 and ****p <0.0001.

In order to study the photothermal toxicity of CroR@aB2MG on senescent cells, we first established a senescent cell model induced by DOX [28, 29]. As shown in Figure 4A, the staining of SA-β-GAL was visible in NHDF cells treated with DOX, and there was an obvious increase in the level of SA-β-GAL expression when the concentration of DOX reached 100 µM, indicating that 100 µM is a suitable concentration for DOX to induce senescence. The successful induction of senescent NHDF cells was also shown by increased levels of senescence-related p16^ink4a^ [30], DcR2 [31], FOXO4 [32] and p53 [33] and a loss of lamin B1 [34] (Fig. 4B-E).

**Figure 5.**
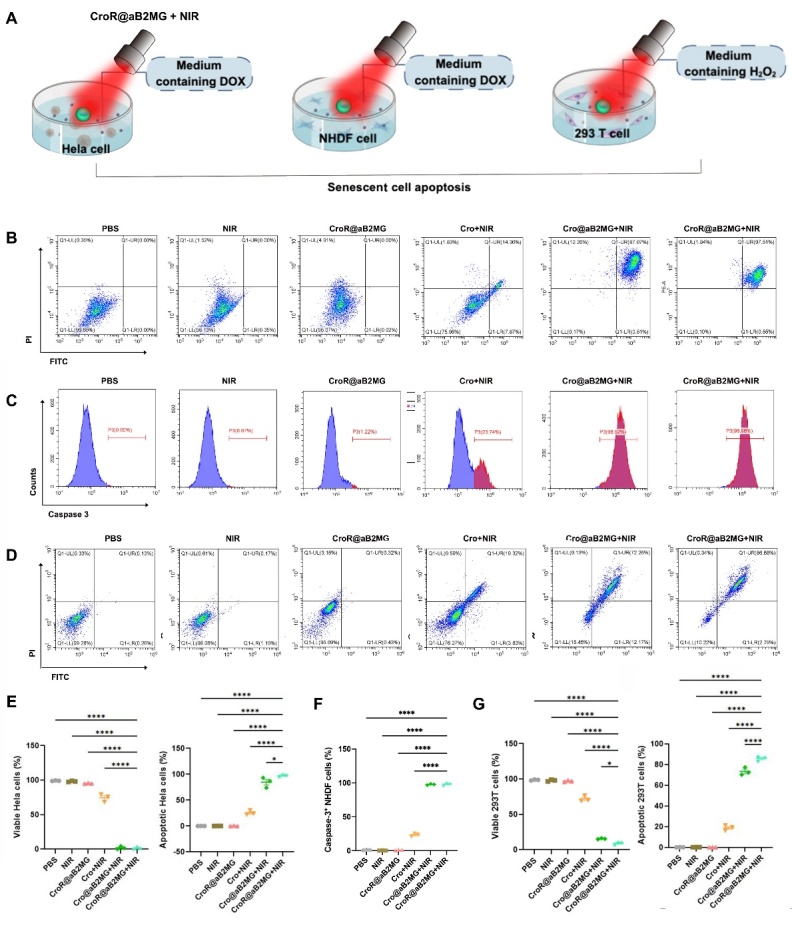
**Photoactivated CroR@aB2MG promotes the apoptosis of chemical-induced senescent cells**. **(A)** A diagram showing the operation of CroR@aB2MG-mediated PTT on DOX-treated Hela cells, DOX-treated NHDF cells, and H_2_O_2_-treated 293 T cells to induce apoptosis. (B, C and D) Representative flow cytometry plots of viable and apoptotic cells in the three types of chemical-induced senescent cells mentioned in (A) after the treatment of PBS, NIR irradiation, CroR@aB2MG, Cro in combination with NIR irradiation (Cro+NIR), Cro@aB2MG in combination with NIR irradiation (Cro@aB2MG+NIR) or CroR@aB2MG in combination with NIR irradiation (CroR@aB2MG+NIR). The apoptotic populations were indicated by the labeling of Annexin V-FITC/PI or caspase 3. In (C), the blue and red spectral bands indicate caspase 3-negative and -positive cells respectively. (E, F and G) The percentages of viable and/or apoptotic cells represented by (B), **(C)** and (D), respectively. N=3 batches of cells indicating 3 biological replicates. The significance of differences in the proportions of cells between different groups was determined through one-way ANOVA with Tukey's *post hoc* test and shown as *p <0.05 and ****p <0.0001.

Meanwhile, we tested the cytotoxicity of CroR@aB2MG on senescent NHDF cells induced by DOX, 293T cells induced by H_2_O_2_ and Hela cells induced by DOX. All three types of induced senescent cells retained high viability even when the concentration of CroR@aB2MG was as high as 1 mg/mL after 24h incubation (Supplementary Fig. S4), indicating high biocompatibility of CroR@aB2MG with living cells. After the treatment of PBS, CroR@aB2MG or NIR irradiation of 0.75 W/cm^2^, the viability of senescent NHDF, 293 T or Hela cells was maintained above 90%, that of cells treated with Cro plus NIR irradiation was decreased to below 77%, and that of cells treated with Cro@aB2MG- or CroR@aB2MG-mediated NIR irradiation was diminished to less than 16% (Fig. 5). Particularly, CroR@aB2MG nanoparticles coupled with NIR irradiation killed nearly all senescent cells shown by Annexin V-FITC/PI staining (97.51% ± 3.14%, 86.68% ± 2.46 %) (Fig. 5B, 5D, 5E and 5G) and a high percentage of caspase 3-positive cells (98.96% ± 1.97%) (Fig. 5C and 5F). These results suggest that photoactivated CroR@aB2MG has great potential for eliminating senescent cells.

**Figure 6.**
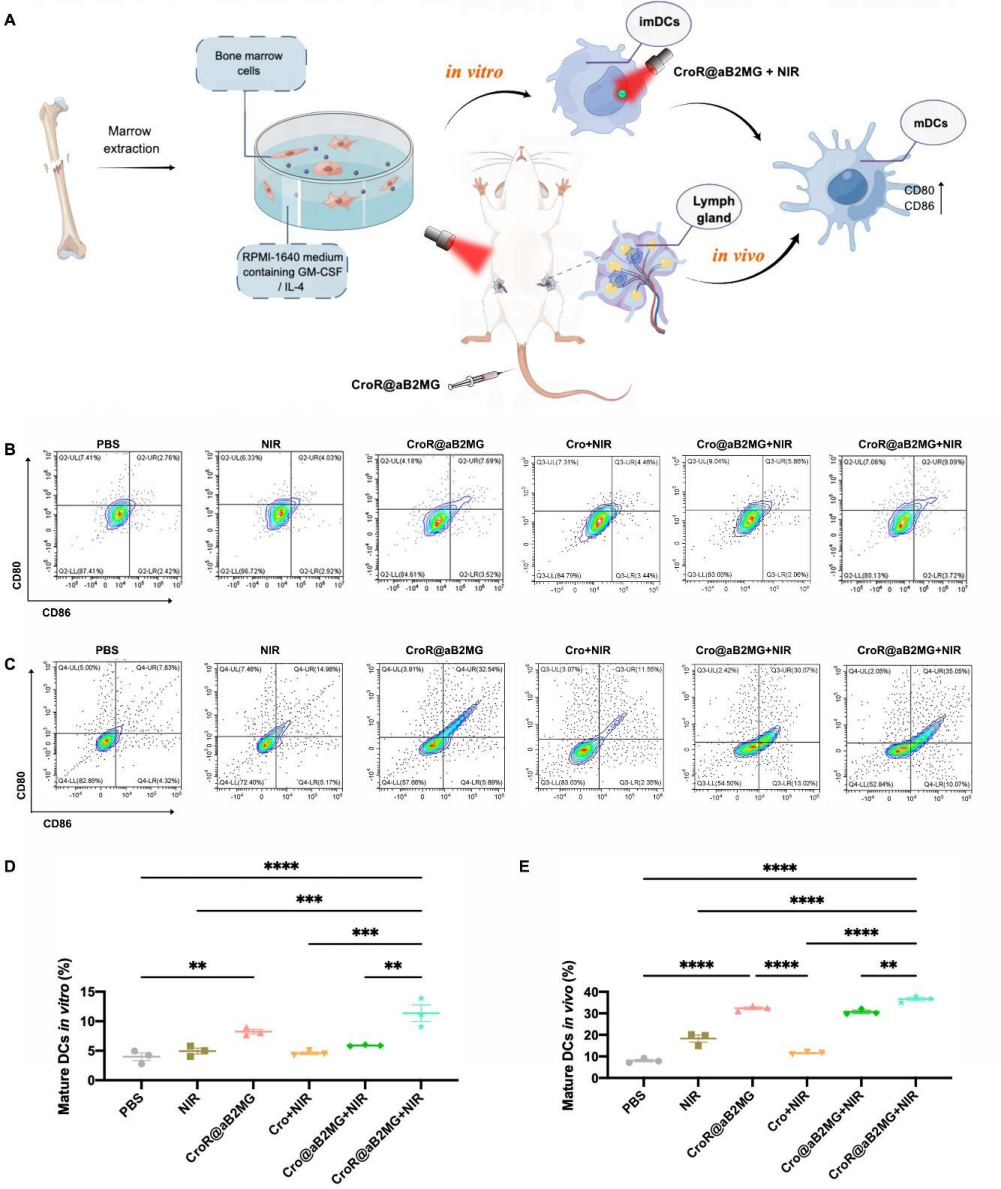
**CroR@aB2MG promotes the maturation of DCs**. **(A)** A diagram showing the promotion of DCs maturation by CroR@aB2MG-mediated PTT *in vitro* and *in vivo*. ImDCs and mDCs indicate immature and mature DCs respectively. (B and D) Representative flow cytometry plots and quantification of mature DCs *in vitro*. The immature DCs cultured *in vitro* were isolated from mouse bone marrow and were treated with PBS, NIR, CroR@aB2MG, Cro+NIR, Cro@aB2MG+NIR and CroR@aB2MG+NIR, respectively. The co-expression of CD80 and CD86 indicates the maturation of DCs. (C and E) Representative flow cytometry plots and quantification of mature DCs *in vivo*. The DCs in DOX-induced senescent mice were collected from their inguinal lymph nodes after different treatments mentioned above. For (D), n=3 batches of cells and for (E), n=3 mice/group, both indicating 3 biological replicates. The significance of differences in the proportions of CD80^+^CD86^+^ DCs between different groups was determined through one-way ANOVA with Tukey's *post hoc* test, and shown as **p <0.01, ***p <0.001, and ****p <0.0001.

**Figure 7.**
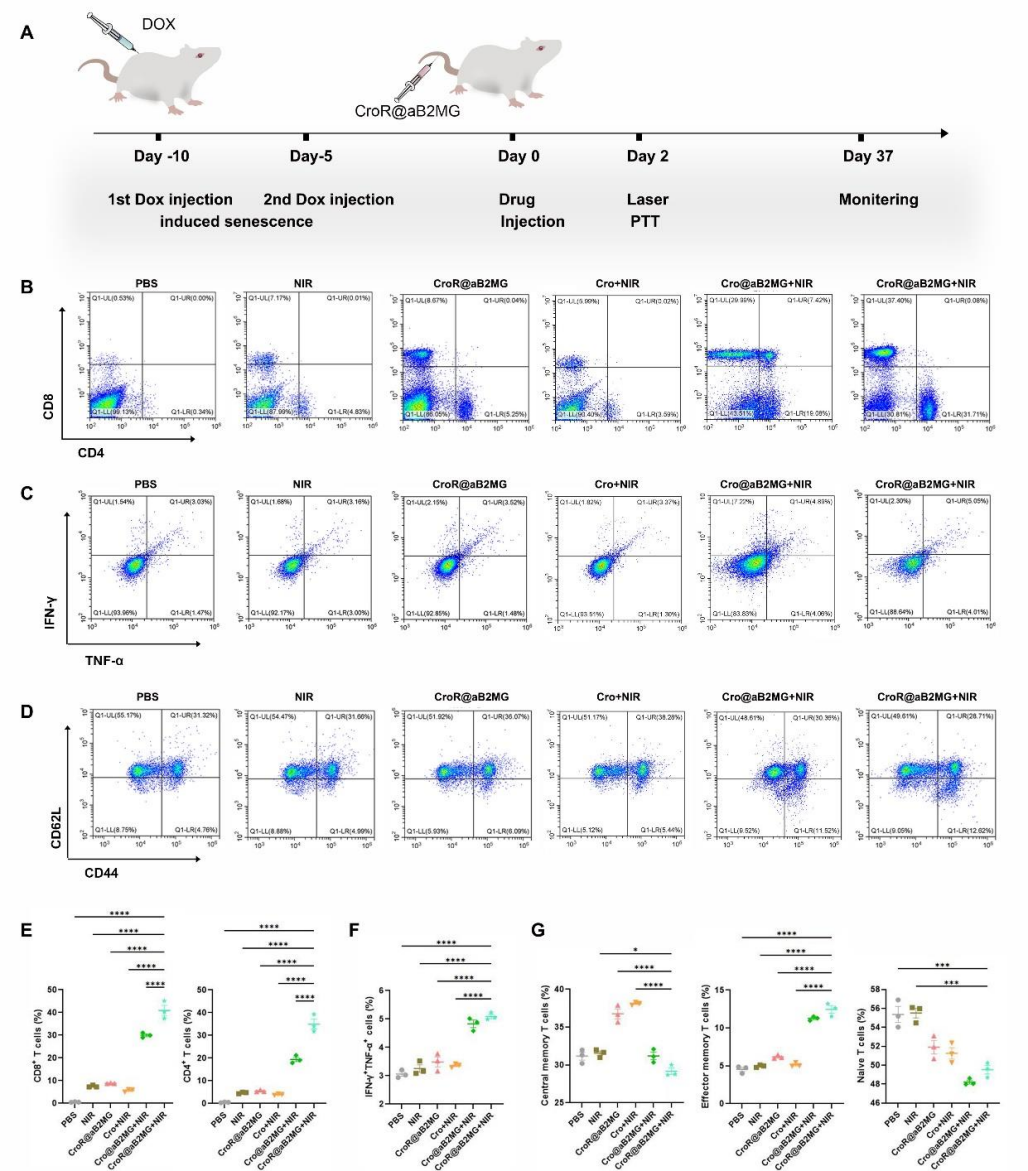
**Photoactivated CroR@aB2MG nanoparticles act as an effective immunopotentiator**. **(A)** Schematic illustration of senescence induction, CroR@aB2MG-mediated PTT and the evaluation timeline in mice. (B, C and D) Representative flow cytometry plots and (E, F and G) quantification of CD8+ T cells, CD4+ T cells, cells co-expressing IFN-γ and TNF-α, central memory T cells (CD62L^high^CD44^high^), effector memory T cells (CD62L^low^CD44^high^) and naive T cells (CD62L^high^ CD44^low^) in the spleens of DOX-induced mice treated with PBS, NIR, CroR@aB2MG, Cro+NIR, Cro@aB2MG+NIR or CroR@aB2MG+NIR. For (E-G), n=3 mice/group, indicating 3 biological replicates. The significance of differences in the proportions of cells between different groups was determined through one-way ANOVA with Tukey's *post hoc* test, and shown as *p <0.05, ***p <0.001, and ****p <0.0001.

### CroR@aB2MG nanoparticles-mediated immune responses in vitro and in vivo

To pinpoint the specific mechanism of photoactivated CroR@aB2MG nanoparticles mediating the clearance of senescent cells, we examined their potential for the activation of immune cells *in vitro*. Among different types of immune cells, DCs present the antigen of an organism invader to T cells and play a key role in initiating and regulating immune responses [35]. DCs were extracted from mouse bone marrow and cultured[36], followed by the co-culture with a specific photothermal nanomaterial (Fig. 6A). Our results showed that compared with PBS, CroR@aB2MG significantly increased the proportion of cells expressing CD80 and CD86, markers of mature DCs [37] (Fig. 6B and 6D). This indicates that CroR@aB2MG can initiate immune responses as expected from the effect of R837. Moreover, the percentage of mature DCs treated with CroR@aB2MG and NIR irradiation together (CroR@aB2MG+NIR) was significantly higher than those treated with NIR irradiation alone, Cro combined with NIR irradiation (Cro+NIR) or Cro@aB2MG combined with NIR irradiation (Cro@aB2MG+NIR), suggesting that CroR@aB2MG-miediated PTT can maximize the promoting effect of DCs maturation *in vitro*.

Next, we further explored if CroR@aB2MG could induce immune activation *in vivo*. We used an aging mouse model with DOX induction (Fig. 6A and 7A). Similar to the observations *in vitro*, compared with the control group or the group injected with Cro, the lymph nodes of DOX-induced aging mice treated with CroR@aB2MG (with or without NIR irradiation) had significantly increased percentages of cells expressing CD80 and CD86 48h after CroR@aB2MG administration (Fig. 6C and 6E). Importantly, the proportions of spleen-derived CD4^+^ T cells and CD8^+^ T cells in the CroR@aB2MG+NIR-treated group were significantly higher than those of any other group (Fig. 7B and 7E). Correspondingly, the percentage of cells co-expressing IFN-γ and TNF-α in the CroR@aB2MG+NIR-treated group increased significantly compared with the control group and groups treated with CroR@aB2MG or NIR alone (Fig. 7C and 7F), indicating a faster immune response. Interestingly, there were more effector memory T cells (CD62L^low^ CD44^high^) and less central memory T cells (CD62L^high^ CD44^high^) in CroR@aB2MG+NIR-treated group compared with groups treated with CroR@aB2MG or NIR irradiation alone (Fig. 7D and 7G). Whereas, the percentage of naive T cells (CD62L^high^ CD44^low^) in CroR@aB2MG+NIR-treated group decreased compared with the control group or the group treated with NIR irradiation alone (Fig. 7D and 7G). These results suggest that the immune responses induced by CroR@aB2MG+NIR cover all stages of the immune clearance process and even include the immediate immunological memory effect.

**Figure 8.**
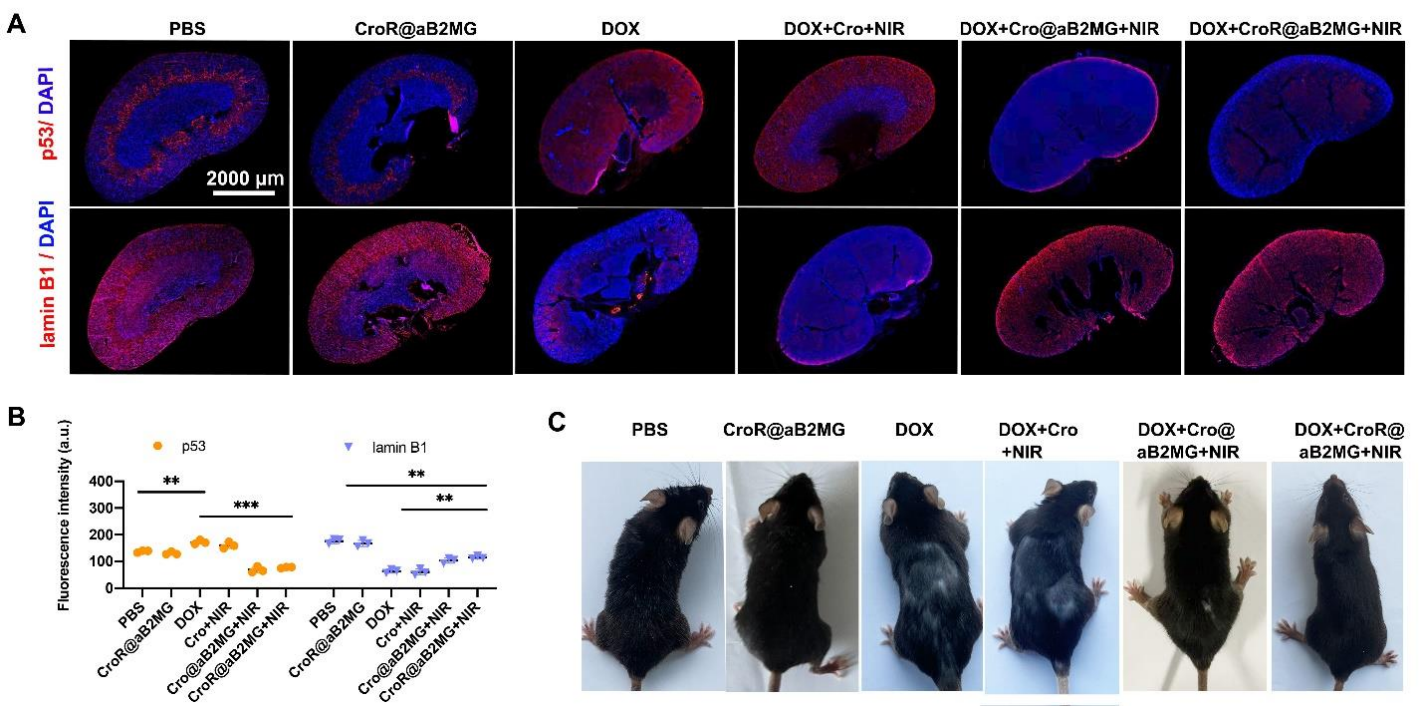
**CroR@aB2MG-mediated PTT effectively relieves senescence *in vivo***. **(A)** Representative images of kidneys from mice treated with PBS, CroR@aB2MG, DOX, DOX+Cro+NIR, DOX+Cro@aB2MG+NIR or DOX+CroR@aB2MG+NIR stained for p53 and lamin B1. **(B)** Quantification of fluorescence intensities of immunolabeling for p53 and lamin B1 in kidney sections of different mouse groups represented by (A). **(C)** Representative images of fur appearance of mice receiving different treatments indicated in (A). For (B), n=3 mice/group. For each mouse, 6 consecutive sections located in the middle of one kidney were taken and subjected to immunostaining, 3 for p53 and the other 3 for lamin B1. For each section, 3 regions of interest were selected randomly as technical replicates and quantified for fluorescence intensities. The 3 intensity values were averaged for one section and the mean values of 3 sections (9 technical replicates in total) were averaged again to obtain a single data point to indicate the expression level of the target protein in one mouse as one replicate. The 3 mice were regarded as 3 biological replicates. The significance of differences in the expression level between different mouse groups was determined through one-way ANOVA with Tukey's *post hoc* test and shown as **p <0.01 and ***p <0.001. For (C), a cohort of at least 5 mice in each group were observed and compared.

### Synergistic effect of PTT-induced apoptosis and CroR@aB2MG for senescence clearance *in vivo*

The experimental results above confirmed that CroR@aB2MG in combination with NIR irradiation selectively killed senescent cells, which prompted us to investigate the effect of CroR@aB2MG on senescence *in vivo*. As expected, the expression level of aging-related protein p53 was significantly increased in the kidney of DOX-treated mice, while that of lamin B1 was significantly decreased (Fig. 8A and 8B).To evaluate the anti-aging effect of NIR-induced apoptosis augmented with senescent cell targeting and immune adjuvant, aged mice were injected with CroR@aB2MG (0.5 mg) through the tail vein and irradiated with an 808nm NIR laser. The combined effects of NIR irradiation and CroR@aB2MG decreased the expression level of p53 and increased that of lamin B1 in mouse kidney 35 days after the treatment (Fig. 8A and 8B). Further, the combination of CroR@aB2MG administration and NIR irradiation increased the fur density, improved the hair loss of DOX-induced senescent mice and showed better recovery effects than Cro+NIR or Cro@aB2MG+NIR (Fig. 8C). These results suggest that photoactivated CroR@aB2MG can significantly improve DOX-induced renal aging in mice. It is also worthwhile to mention that CroR@aB2MG alone changed neither the expression levels of p53 and lamin B1 nor the fur appearance of DOX-induced mice, suggesting its high biosafety *in vivo*.

## DISCUSSION

In the progression of aging, senescent cells can be found in multiple organs due to various external and internal stress stimuli. These cells enter a state of permanent growth arrest, but still remain metabolically active. Unlike normal cells that secrete necessary cytokines and signaling molecules to maintain the regular functioning of tissues and organs, senescent cells produce a more complex secretome including SASPs that may induce inflammation and reinforce senescence by influencing nearby cells [38]. While temporary accumulation of SASPs elicits immune responses and initiates immune clearance, persistent secretion of SASPs disrupts the immune homeostasis and impairs the self-repair capacity of the organism [39]. Due to the pleiotropic effects of SASPs, therapeutic strategies targeting senescent cells have become increasingly refined for treating chronic diseases. For example, apart from the traditional radiotherapy and chemotherapy that can cause tumor senescence, the induction of SASPs secretion may enhance immune surveillance for the clearance of senescent cancer cells and modify the tumor microenvironment to facilitate the exposure to antitumor treatments [40]. Meanwhile, as both senescent cells and SASPs are highly heterogeneous and may hold beneficial effects, the selection of proper senescence markers for precise targeting is crucial. Combining the afore-mentioned therapeutic concepts, we designed an all-inclusive photoactivated nanoparticle CroR@aB2MG to target senescent cells with an anti-B2MG antibody, accelerate the apoptosis of senescent cells through NIR irradiation and initiate immune clearance with immune adjuvant R837. To our knowledge, the augmented cell clearance strategy was seldom reported for the treatment of aging and aging-related diseases to date.

PTT is a widely adopted approach that uses the heat generated from the light mostly in the wavelengths of NIR to ablate harmful cells and tissues in various diseases including cancer. The efficient realization of PTT largely relies on the photothermal conversion efficiency of the photosensitizing agents. Also, Nomura and colleagues showed that tumor cure requires a heating temperature of 43^o^C and proposed against overheat such as 50^o^C to avoid the necrosis of the normal tissue [41]. The CroR@aB2MG nanoparticles we synthesized meet this requirement in 5 min with a photothermal conversion efficiency of 70.4%, promising an ideal choice of photothermal agent.

The highly efficient inorganic photothermal agents [42] including noble metal nanoparticles, semiconductor nanomaterials and carbon-based nanomaterials have low biocompatibility and biodegradability and are therefore limited in clinical applications. In this regard, cyanine-, diketopyrrolopyrrole-, croconaine-, porphyrin-, poly-aniline-, polypyrrole- and other small molecules-based organic photothermal agents [43] are more commonly preferred in the development of clinically translatable drugs. In this research, we selected the highly photostable and flexible croconium dye (croconaine) [44], which is widely used as an NIR agent. Croconium dyes, in the form of nanoparticles, accumulate well in tumor and undergo rapid clearance with minimal toxicity to the normal tissue [45, 46]. Indeed, we showed that CroR@aB2MG administration alone did not change the renal senescence or the fur appearance of normal mice, suggesting a high level of biosafety.

CroR@aB2MG targets senescent cells effectively, with the direction of an anti-B2MG antibody. The expression of blood-borne B2MG, a biomarker for cancer and many inflammatory diseases [47-49] has been found positively correlated with age, the levels of p16^ink4a^, IL-1β and IL-6, and the oxidative stress [50, 51]. This is in line with our results, as CroR@aB2MG-mediated NIR irradiation reduced DOX-boosted senescence marked by the enhanced expression of p16^ink4a^, p53, DcR2, FOXO4 and the loss of lamin B1. Importantly, aged but not young B2mg knockout mice exhibit improved cognitive function and neurogenesis [52]. Cigarette smoke extract-induced senescence and growth arrest of alveolar epithelial cells are associated with increased expression of B2MG and can be partly blocked by an anti-B2MG antibody *in vitro* [53]. In addition, Zhang and colleagues reported that the up-regulation of B2MG expression in gliomas might lead to immunosuppression by interacting with immune checkpoint molecules in the tumor microenvironment [47]. Since CroR@aB2MG is highly efficient in targeting B2MG-expressing cells, it can be applied to systematically reduce the circulating B2MG levels *in vivo*, introduce local ablation of damaged tissue and ameliorate the side effects of immunotherapy to the normal tissue. We propose that CroR@aB2MG-integrated PTT may provide further insights into the development of novel therapeutic approaches for neurodegenerative diseases, lung diseases and cancer.

In order to ensure optimum prognosis, strategies of strengthening the immune system are being developed to control the recurrence possibilities of chronic diseases. Nanomedicine has been proposed as a paradigm to regulate trained immunity by interacting with phagocytic myeloid cells [54]. CroR@aB2MG nanoparticles, in combination with NIR irradiation are capable of stimulating the three major types of immune cells in the whole process of immune clearance, which are mature DCs that present senescent cells damaged by NIR irradiation, CD4^+^ helper T cells that release immune cytokines such as IFN-γ and TNF-α, and cytotoxic CD8^+^ T cells that execute the killing of damaged cells. Particularly, the percentage of effector memory T cells that exert immediate protective memory function was increased. Correspondingly, the proportions of central memory T cells and naive T cells were decreased. As central memory T cells that mediate non-immediate reactive memory may differentiate into effector cells when activated by antigens [55], our results indicate that CroR@aB2MG nanoparticles are capable of evoking immunological memory and remodeling the adaptive immune system. These immune effects are triggered by immune adjuvant R837, which generated similar immune responses in another type of nanoparticle HMME/ R837@Lip for preventing tumor relapse [56].

To further validate the therapeutic potential of our photoactivated clearance strategy, we propose to study the local and systematic anti-aging effects of CroR@aB2MG-integrated PTT in naturally aging animals and animal models of age-related diseases exampled by neurodegenerative diseases, chronic obstructive pulmonary disease and cancer, and next in samples (cells and tissues) collected from elderly individuals and patients suffering from the aforementioned diseases as pre-clinical research models *in vitro*. We expect that this new therapeutic strategy may provide a universal approach for therapies against multiple senescence-associated diseases using nanomaterials.

## Supplementary Materials

The Supplementary data can be found online at: www.aginganddisease.org/EN/10.14336/AD.2023.0628-1.


