# Supplementary material for: An Antibody-directed and Immune Response Modifier-augmented Photothermal Therapy Strategy Relieves Aging via Rapid Immune Clearance of Senescent Cells
Source: Aging Dis. 2024 Apr 1;15(2):787–803. doi: 10.14336/AD.2023.0628-1 (PMC10917526; doi:10.14336/AD.2023.0628-1)
Supplement: Supplementary file 1 [file AD-15-2-787-s.pdf]

SUPPLEMENTARY DATA

An Antibody-directed and Immune Response Modifier-augmented Photothermal Therapy Strategy Relieves Aging via Rapid Immune Clearance of Senescent Cells

Jiamei Han, Judun Zheng, Qian Li, Huanle Hong, Jing Yao, Jiao Wang, Robert Chunhua Zhao

© 2023. Han J et al. Published online at http://www.aginganddisease.org/EN/10.14336/AD.2023.0628-1

SUPPLEMENTARY DATA

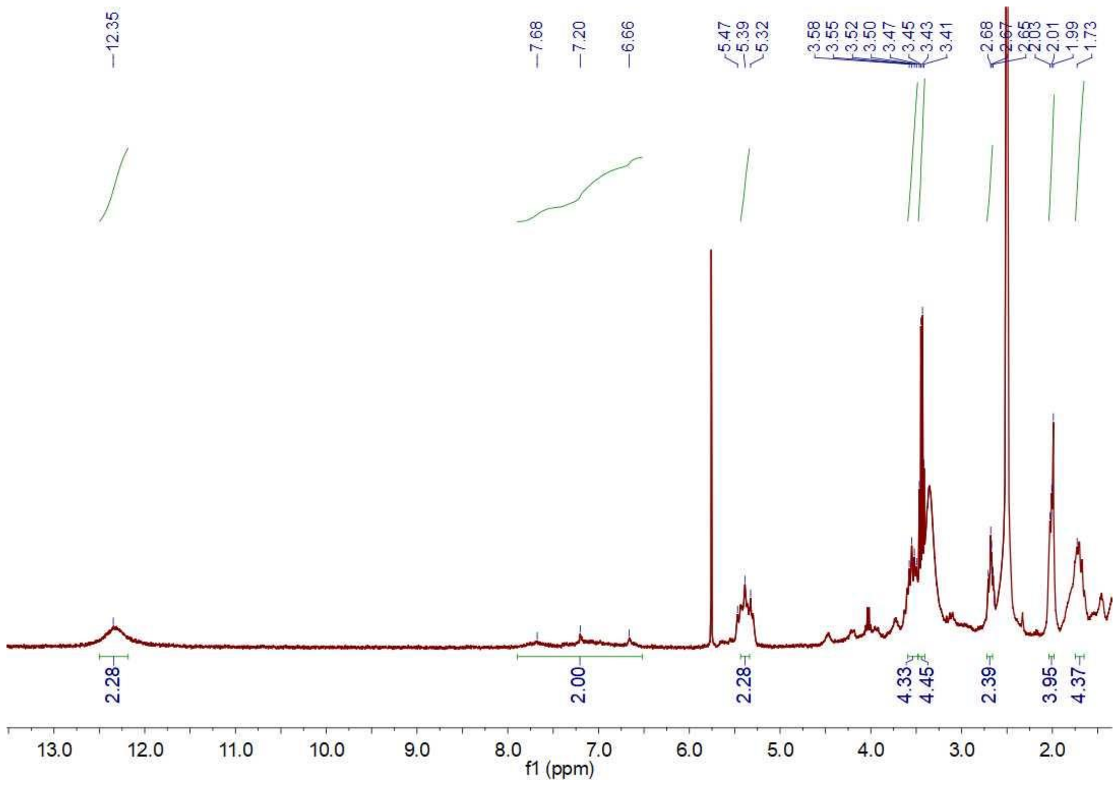

Supplementary Figure 1. The ^{1}H NMR spectrum of the synthesized Cro dye.

© 2023. Han J et al. Published online at http://www.aginganddisease.org/EN/10.14336/AD.2023.0628-1

SUPPLEMENTARY DATA

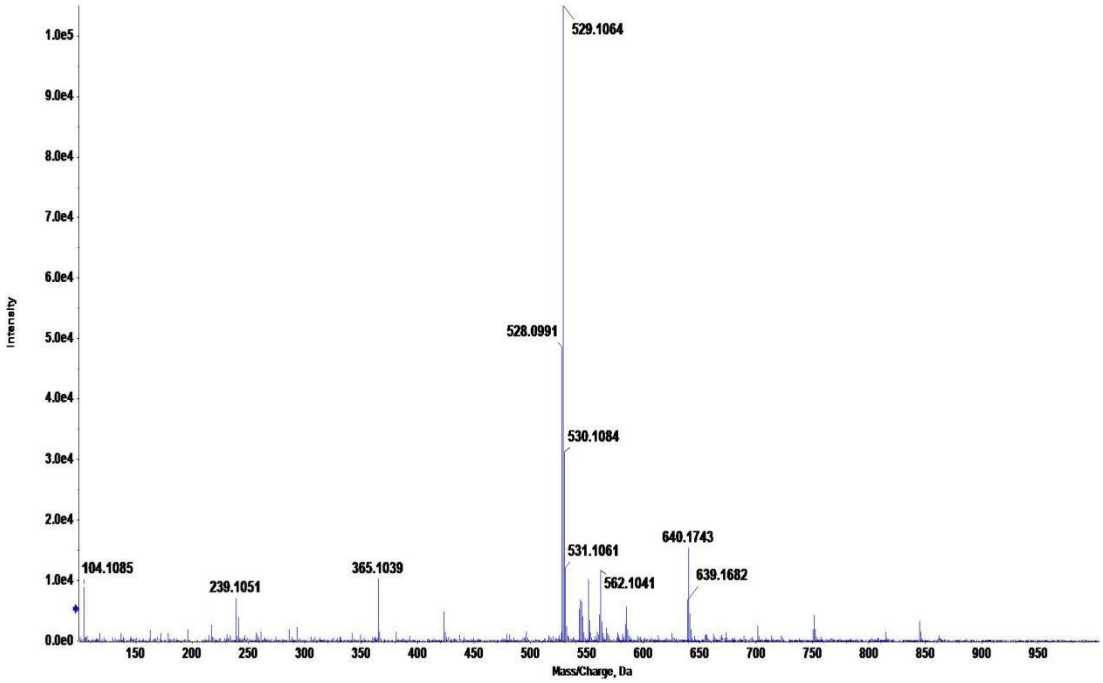

Supplementary Figure 2. The ESI-MS spectrum of the Cro dye.

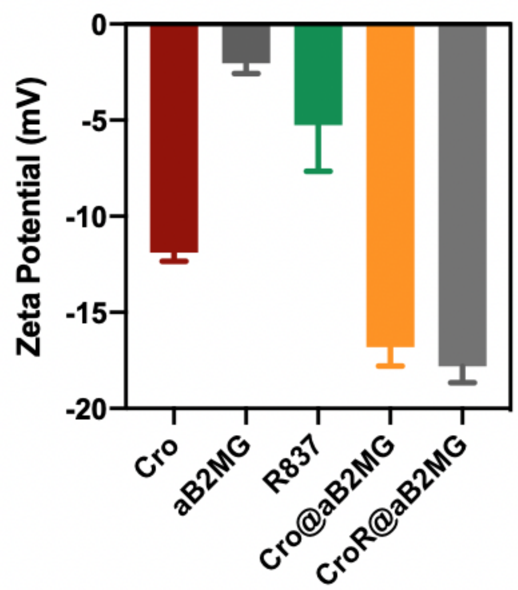

Supplementary Figure 3. The zeta potentials of Cro, aB2MG, R837, Cro@aB2MG and CroR@aB2MG. The values were quantified from 3 independent replicates.

© 2023. Han J et al. Published online at http://www.aginganddisease.org/EN/10.14336/AD.2023.0628-1

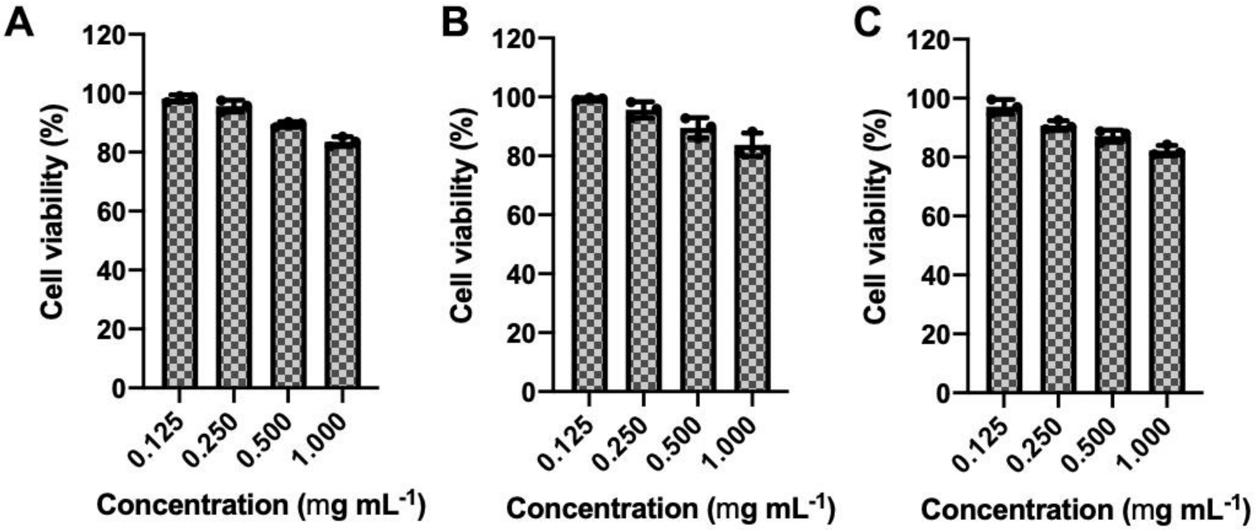

Supplementary Figure 4. The viability of (A) DOX-induced senescent NHDF cells, (B) H_2O_2-induced senescent 293T cells, and (C) DOX-induced senescent Hela cells incubated with various concentrations of CroR@aB2MG. A CCK-8 kit was used to determine cell viability. The values were quantified from 3 batches of cells as 3 biological replicates for each cell type.

© 2023. Han J et al. Published online at http://www.aginganddisease.org/EN/10.14336/AD.2023.0628-1
